# Time toxicity of intravesical BCG versus sequential intravesical gemcitabine and docetaxel for non‐muscle‐invasive bladder cancer

**DOI:** 10.1002/bco2.70215

**Published:** 2026-05-14

**Authors:** Jacob Hanna, Melinda Z. Fu, Berk Inan, David Guevara, Lucille Lannan, Disha Patel, Mahdi Hemmati Ghavshough, David Golombos, Thomas Jang, Saum Ghodoussipour, Vignesh T. Packiam

**Affiliations:** ^1^ Rutgers Robert Wood Johnson Medical School New Brunswick New Jersey USA; ^2^ Section of Urologic Oncology Rutgers Cancer Institute New Brunswick New Jersey USA

**Keywords:** BCG, bladder cancer, docetaxel, gemcitabine, intravesical therapy, non–muscle‐invasive bladder cancer, time toxicity

## Abstract

**Objectives:**

This study aimed to evaluate the time toxicity of intravesical BCG compared to sequential intravesical gemcitabine and docetaxel (Gem/Doce) for non–muscle‐invasive bladder cancer (NMIBC).

**Patients and Methods:**

After IRB approval, we retrospectively reviewed patients with NMIBC treated with BCG or Gem/Doce between 2022 and 2025 at our institution. Medical records were used to calculate time for intravesical therapy, office visits, transurethral resection of bladder tumour (TURBT) and emergency department encounters. Continuous variables were compared using the Wilcoxon rank sum test with Hodges–Lehmann estimates.

**Results:**

Of 133 patients, 86 received BCG (65%) and 47 Gem/Doce (35%). Patients with BCG were more likely treatment naïve compared to those with Gem/Doce (77% vs 55%, *p* = 0.018). Median time per instillation was 179 min for BCG compared to 192 min for Gem/Doce (Hodges–Lehmann shift +27 min, 95% CI 4 to 57; *p* = 0.02). Median TURBT time, office visit time and emergency department visit time were similar between groups (all *p* > 0.05). Over the first six instillations, the cumulative difference was 306 min (+5.1 h, *p* < 0.001).

**Conclusion:**

Gem/Doce requires a greater time investment than BCG. As effective NMIBC therapies expand, time toxicity should be considered alongside oncologic outcomes.

## 1 INTRODUCTION

Non–muscle‐invasive bladder cancer (NMIBC) constitutes 75% of new bladder cancer diagnoses and requires intensive surveillance and often repeated intravesical therapy, placing a considerable burden on patients and health systems.^1^, ^2^ Bacillus Calmette–Guérin (BCG) remains the cornerstone intravesical agent for intermediate and high‐risk NMIBC, providing durable disease control and reducing the risk of progression.^3^, ^4^ However, sustained global BCG shortages have challenged consistent delivery of this standard therapy across institutions.^5^

Novel intravesical therapies have emerged due to the BCG shortage and need for effective salvage bladder sparing options in the BCG unresponsive space. One increasingly utilised regimen is sequential intravesical gemcitabine and docetaxel (Gem/Doce). Data for Gem/Doce were first published in 2015.^6^ Retrospective data have demonstrated promising oncologic outcomes in both BCG‐naïve and BCG‐unresponsive cohorts, with recurrence‐free survival rates mirroring those historically reported for BCG.^7^, ^8^, ^9^, ^10^ Several single‐centre and multi‐institutional series have also suggested favourable tolerability and completion rates, leading to increased real‐world adoption and ongoing randomised trials directly comparing Gem/Doce with BCG.^11^, ^12^ Although these results are encouraging, Gem/Doce administration does portend a significant treatment burden on patients and the healthcare system.

Time toxicity has been proposed as a complementary endpoint to financial toxicity and quality of life in oncology, capturing the cumulative hours patients devote to clinic visits, procedures, hospitalisations and recovery, all of which may influence treatment preference, satisfaction and adherence.^13^, ^14^, ^15^ No study to our knowledge has quantified the time burden associated with BCG versus Gem/Doce.

The present study addresses this gap by examining the healthcare time experienced by patients with NMIBC receiving BCG or Gem/Doce at a single academic centre.

## 2 METHODS

After obtaining approval from the institutional review board, we performed a retrospective cohort study at Rutgers Robert Wood Johnson University Hospital including all patients with NMIBC treated with either Gem/Doce or BCG between January 2022 and March 2025. A waiver of informed consent was granted. Clinicopathologic features including age, sex, tumour stage and treatment history were abstracted from the electronic health record. Hospitalisations, defined as emergency department visits or inpatient admissions, after intravesical therapy treatment began, were recorded. Reasons for presentation were classified as hematuria, infection, acute kidney injury, gastrointestinal complications or other. Admissions clearly unrelated to bladder cancer treatment, such as mechanical falls, chronic obstructive pulmonary disease exacerbations, or congestive heart failure admissions, were excluded. Hospital length of stay was not analysed because admission duration was highly heterogeneous.

Gem/Doce and BCG were administered as previously described.^16^ Notably, in our institution, there is a low threshold to keep patients in the office if there are concerns of adequate BCG retention with the catheter removed.

The primary outcome was per‐encounter treatment time, examining the duration of individual instillations, transurethral resection of bladder tumours (TURBTs) or office visits between BCG and Gem/Doce groups. Treatment times were defined in a patient‐centric manner as follows. For TURBT, time was recorded from hospital check‐in to discharge rather than operative time. Only TURBTs performed at our institution were included in TURBT‐specific analyses, with no assumptions or imputations made for missing encounters. For office visits, time was recorded from initial nursing interaction (vital signs or consent) to when after‐visit summaries were printed and given to the patient. For both BCG and Gem/Doce instillations, time was recorded from documentation of consent or vital signs to catheter removal, as recorded in nursing notes. These differences were preserved in analysis to reflect actual patient time commitments and not protocolised estimates. For all encounter‐level analyses, only visits with complete start and end times were included; incomplete data were excluded rather than estimated.

A standardised induction analysis was performed comparing the cumulative time of the first six instillations between groups. A prespecified per‐protocol sensitivity analysis was performed restricting to patients completing at least five instillations.

Continuous variables were summarised as medians with interquartile ranges and compared between groups using the Wilcoxon rank‐sum test. Effect sizes were estimated using Hodges–Lehmann median shifts with 95% confidence intervals. Categorical variables were summarised as counts and percentages and compared with chi‐square or Fisher's exact tests, as appropriate. A two‐tailed *α* of 0.05 was considered statistically significant. Analyses were performed using SAS version 9.4 (SAS Institute, Cary, NC, USA).

## 3 RESULTS

A total of 133 patients were included in this analysis, of whom 86 (65%) received BCG and 47 (35%) received Gem/Doce. Baseline demographic and clinical characteristics were generally similar between groups. The median age was 73 years in the BCG cohort and 74 years in the Gem/Doce cohort (*p* = 0.72). Sex distribution did not differ significantly, with 73% male patients in the BCG group and 74% in the Gem/Doce group (*p* = 0.88). Tumour stage distributions were similar between groups, with T1 disease in 51% of BCG patients and 49% of Gem/Doce patients, Ta disease in 30% and 21%, respectively, and Tis in 19% and 30%, respectively (*p* = 0.27). However, patients in the Gem/Doce cohort were significantly more likely to be previously treated, with 45% classified as non‐naïve compared to 23% in the BCG cohort (*p* = 0.018) (Table 1).

**TABLE 1:** Baseline patient characteristics and treatment outcomes.

Characteristic	BCG (*n* = 86)	Gem/Doce (*n* = 47)	*p*‐value
Demographics			
Age, years, median (IQR)	73 (67, 80)	74 (69, 80)	0.72
Male sex, *n* (%)	63 (73)	35 (74)	0.88
Disease characteristics			
Tumour stage, *n* (%)			0.27
T1	44 (51)	23 (49)	
Ta	26 (30)	10 (21)	
Tis	16 (19)	14 (30)	
Treatment‐naïve, *n* (%)	66 (77)	26 (55)	0.018
Safety and treatment completion			
Hospitalisation, *n* (%)	26 (30)	11 (23)	0.40
Completed ≥5 induction instillations, *n* (%)	78 (91)	45 (96)	0.29

Abbreviations: BCG, bacillus Calmette–Guérin; Gem/Doce, sequential intravesical gemcitabine and docetaxel; IQR, interquartile range.

### 3.1 Per‐encounter treatment times

Median instillation time differed between groups, with Gem/Doce, at 192 min (IQR 174, 215) compared to 179 min (IQR 97, 213) with BCG, a Hodges–Lehmann median shift of +27 min (95% CI 4, 57; *p* = 0.02). In contrast, median total encounter time for TURBT duration did not differ significantly between groups, with patients in the BCG cohort at a median of 431 min (IQR 363, 510; *n* = 69) versus 400 min (IQR 360, 450; *n* = 47) for those receiving Gem/Doce (*p* = 0.07). Similarly, office visits were comparable, with median durations of 82 min (IQR 57, 133; *n* = 80) for BCG and 83 min (IQR 58, 110; *n* = 45) for Gem/Doce (*p* = 0.44) (Table 2).

**TABLE 2:** Time toxicity outcomes.

Outcome	BCG	Gem/Doce	HL Shift (95% CI)	*p*‐value
Per‐instillation time				
Time per instillation, min	179 (97, 213) (3.0 h)	192 (174, 215) (3.2 h)	+27 (4, 57)	0.02
Per‐noninstillation visits				
Time per TURBT, min	431 (363, 510) (7.2 h)	400 (360, 450) (6.7 h)	−32 (−67, 3)	0.07
Time per office visit, min	82 (57, 133) (1.4 h)	83 (58, 110) (1.4 h)	−7 (−25, 10)	0.44
First six instillations				
First 6 instillations, min	845 (580, 1264) (14.1 h)	1188 (1047, 1347) (19.8 h)	+306 (+5.1 h)	<0.001

*Note*: Data presented as median (interquartile range). Hours shown in brackets for interpretability. The Hodges–Lehmann (HL) shift represents the estimated difference (Gem/Doce minus BCG); positive values indicate longer times with Gem/Doce. Statistical comparisons used Wilcoxon rank‐sum tests.

Abbreviations: BCG, bacillus Calmette‐Guérin; CI, confidence interval; Gem/Doce, sequential intravesical gemcitabine and docetaxel; HL, Hodges–Lehmann; TURBT, transurethral resection of bladder tumour.

### 3.2 First six instillations

**FIGURE 1:**
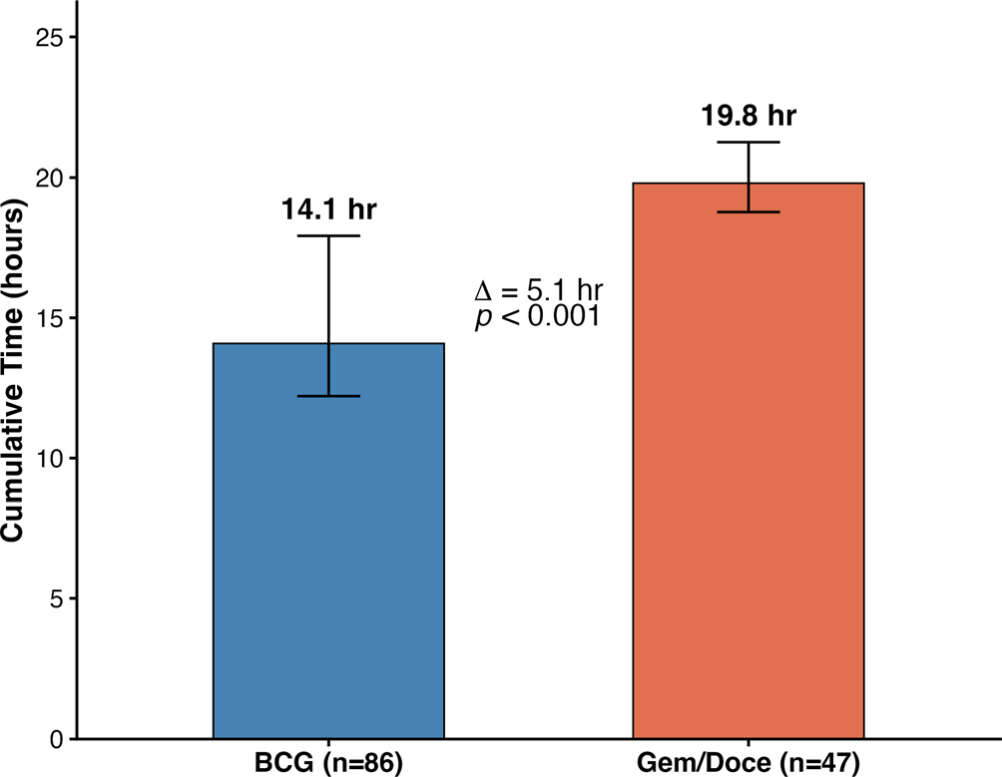
Cumulative time for the first six instillations comparing BCG and Gem/Doce cohorts. Box plots display the median and interquartile range for each instillation. The Hodges–Lehmann estimated shift was +306 min (+5.1 h; *p* < 0.001).

Comparing the first six instillations between groups, the Gem/Doce cohort required 1188 min (IQR 1047, 1347) compared to 845 min (IQR 580, 1264) with BCG, a Hodges–Lehmann estimated shift of 306 min or 51 min per instillation (*p* < 0.001) (Figure 1).

### 3.3 Safety and treatment completion

Hospitalisation rates were similar between groups. Hospitalisation occurred in 30% of patients in the BCG cohort and 23% in the Gem/Doce cohort (*p* = 0.40). Hospitalisation causes differed modestly, with patients in the BCG cohort more often presenting for hematuria or bleeding and those in the Gem/Doce cohort more often for fever, gastrointestinal symptoms or renal complications, although statistical testing was limited by a small number of events. Treatment completion rates were high in both cohorts. At least five instillations were completed by 91% of patients in the BCG cohort and 96% in the Gem/Doce cohort (*p* = 0.29).

### 3.4 Sensitivity analyses

In the per‐protocol cohort restricted to patients completing at least five instillations, Gem/Doce instillations required a median of 35 min longer than BCG (*p* = 0.004).

## 4 DISCUSSION

In this retrospective cohort study, we report the first quantification of the time toxicity of BCG versus Gem/Doce for the management of NMIBC. The high recurrence rate of NMIBC results in intensive surveillance and repeated treatments. The landscape of intravesical therapy for NMIBC continues to expand, with several new agents emerging as alternatives to BCG. As options multiply, clinicians must counsel patients on the tradeoffs of each regimen so that treatment decisions align with individual goals and priorities. Understanding the time toxicity of NMIBC therapies is essential for this patient‐centred approach. Our findings demonstrate that Gem/Doce and BCG differ in per‐instillation encounter times by approximately 27 min per treatment. This resulted in 5.1 h across the first six instillations for treatment induction.

The longer Gem/Doce instillation time reflects the supervised, two‐step administration of this regimen, where patients are required to remain in clinic for 1 h of gemcitabine dwell and sometimes remain in clinic for 1 h of docetaxel dwell as well. Notably, this already represents a reduction from the original Iowa protocol, which called for 90 min of gemcitabine dwell and 90–120 min of docetaxel dwell.^6^, ^7^ The transition to a 1‐h regimen was driven by real world feasibility rather than rigorous pharmacokinetic study, suggesting that time burden is already shaping clinical practice even in the absence of formal comparative data. Despite this time difference in treatment delivery, both patient groups had similar tolerability and safety outcomes. This suggests that the finding of time difference is not correlated with increased toxicity or higher complication rates but is rather associated with therapy administration.

Patients undergoing intravesical therapy often face some of the highest visit frequencies in urologic oncology, with substantial implications for employment, transportation, caregiver burden and overall quality of life. Prior work has shown that treatment protocols for high‐risk NMIBC can extend beyond 3 years, and while symptom burden is highest during induction, quality of life concerns persist throughout the surveillance period.^17^, ^18^ Although the additional 27 min per Gem/Doce instillation accumulates over time, the total added time (5–6 h over the course of 6 weeks) is modest, especially when compared against other real‐world considerations. For instance, ongoing global BCG shortages continue to disrupt timely care. In such circumstances, the availability of an effective alternative may outweigh the modest increased time required for Gem/Doce instillation. From an operational perspective, the median 27‐min increase per instillation remains manageable within most clinical workflows, though this additional chair time may limit the number of patients who can be treated per day.

Our findings provide practical information that can inform shared decision making between patients and clinicians. As the number of intravesical therapy options continues to grow, patients must weigh not only oncologic efficacy and side effect profiles but also the time commitments associated with each regimen. Quantifying time toxicity allows for more transparent counselling and helps patients select treatments that align with their values and life circumstances.

These findings align with a growing body of literature on time toxicity in oncology. Rocque et al.^19^ quantified healthcare‐related time costs in metastatic breast cancer, while Gupta et al.^20^ analysed the CCTG CO.17 colorectal cancer trial and found that time spent in healthcare contact may offset or even exceed the survival benefit conferred by treatment. Such data underscore the importance of discussing time burden when counselling patients, particularly when treatment benefits are modest. Our study extends this concept to NMIBC intravesical therapy, where visit frequency is among the highest in urologic oncology.

Strengths of this study include comprehensive time‐capture from the patient perspective, preservation of real‐world treatment workflows, and robust sensitivity analyses confirming the significance of our findings across subgroups. However, the study was limited by its single‐centre and retrospective design. Furthermore, dwell‐time differences due to institutional protocols may limit generalizability to centres that manage BCG dwell time differently. In particular, practices where most patients are instilled BCG and instructed to void outside the office will show more significant differences between BCG and Gem/Doce time toxicity. Future studies should aim to include multiple centres with different intravesical therapy protocols to validate these findings.

## 5 CONCLUSIONS

In conclusion, Gem/Doce and BCG differ in time toxicity, primarily due to supervised dual‐agent instillation protocols. This time difference of approximately five additional hours over 6 weeks should be balanced by other factors such as emerging prospective oncologic data, other treatment availability (BCG), patient preference and treatment tolerability or institutional constraints. As NMIBC treatment involves frequent procedures and follow‐up visits, the time patients spend engaged in healthcare, termed ‘time toxicity’, represents an important yet understudied dimension of treatment burden. Characterising and understanding the time toxicity of intravesical therapies will allow patients to better understand the trade‐offs of bladder sparing therapies and ensure that treatment decisions are guided by patient goals.

## AUTHOR CONTRIBUTIONS

**Jacob Hanna and Melinda Z. Fu:** Conceptualization; methodology; data curation; writing—original draft; writing—review and editing. **Berk Inan, David Golombos, Lucille Lannan, Disha Patel, and Mahdi Hemmati Ghavshough:** Data curation; writing—review and editing. **Thomas Jang:** Writing—review and editing. **Saum Ghodoussipour and Vignesh T. Packiam:** Supervision; methodology; writing—review and editing.

## CONFLICT OF INTEREST STATEMENT

The following declarations of interests are made by all authors: Jacob Hanna, BS: None. Melinda Z. Fu, MD: None. Berk Inan, BS: None. David Guevara, BA: None. Lucille Lannan, BS: None.

Disha Patel, BA, MS: None. Mahdi Hemmati Ghavshough, MD: None. David Golombos, MD: None. Thomas Jang, MD: None. Saum Ghodoussipour, MD: Speaker/Consultant for Natera, Urogen, Janssen. Vignesh T. Packiam, MD: Speaker/Consultant for Photocure, Veracyte, Valar Labs, Ferring, Janssen. None of the declared interests are directly related to the work described in this manuscript.

## DISCLOSURES

J.H., M.Z.F., B.I., D.Gu, L.L., D.P., M.H.G., D.Go, T.J.—none. SG—Speaker/Consultant for Natera, Urogen, Janssen. VTP—Speaker/Consultant for Photocure, Veracyte, Valar Labs, Ferring and Janssen.

## Data Availability

The data that support the findings of this study are available from the corresponding author upon reasonable request.
